# Potent and Selective Triazole-Based Inhibitors of the Hypoxia-Inducible Factor Prolyl-Hydroxylases with Activity in the Murine Brain

**DOI:** 10.1371/journal.pone.0132004

**Published:** 2015-07-06

**Authors:** Mun Chiang Chan, Onur Atasoylu, Emma Hodson, Anthony Tumber, Ivanhoe K. H. Leung, Rasheduzzaman Chowdhury, Verónica Gómez-Pérez, Marina Demetriades, Anna M. Rydzik, James Holt-Martyn, Ya-Min Tian, Tammie Bishop, Timothy D. W. Claridge, Akane Kawamura, Christopher W. Pugh, Peter J. Ratcliffe, Christopher J. Schofield

**Affiliations:** 1 Chemistry Research Laboratory, Department of Chemistry, University of Oxford, Oxford, United Kingdom; 2 Centre for Cellular and Molecular Physiology, University of Oxford, Oxford, United Kingdom; 3 Target Discovery Institute, University of Oxford, Oxford, United Kingdom; University of Dundee, UNITED KINGDOM

## Abstract

As part of the cellular adaptation to limiting oxygen availability in animals, the expression of a large set of genes is activated by the upregulation of the hypoxia-inducible transcription factors (HIFs). Therapeutic activation of the natural human hypoxic response can be achieved by the inhibition of the hypoxia sensors for the HIF system, i.e. the HIF prolyl-hydroxylases (PHDs). Here, we report studies on tricyclic triazole-containing compounds as potent and selective PHD inhibitors which compete with the 2-oxoglutarate co-substrate. One compound (**IOX4**) induces HIFα in cells and in wildtype mice with marked induction in the brain tissue, revealing that it is useful for studies aimed at validating the upregulation of HIF for treatment of cerebral diseases including stroke.

## Introduction

**Fig 1:**
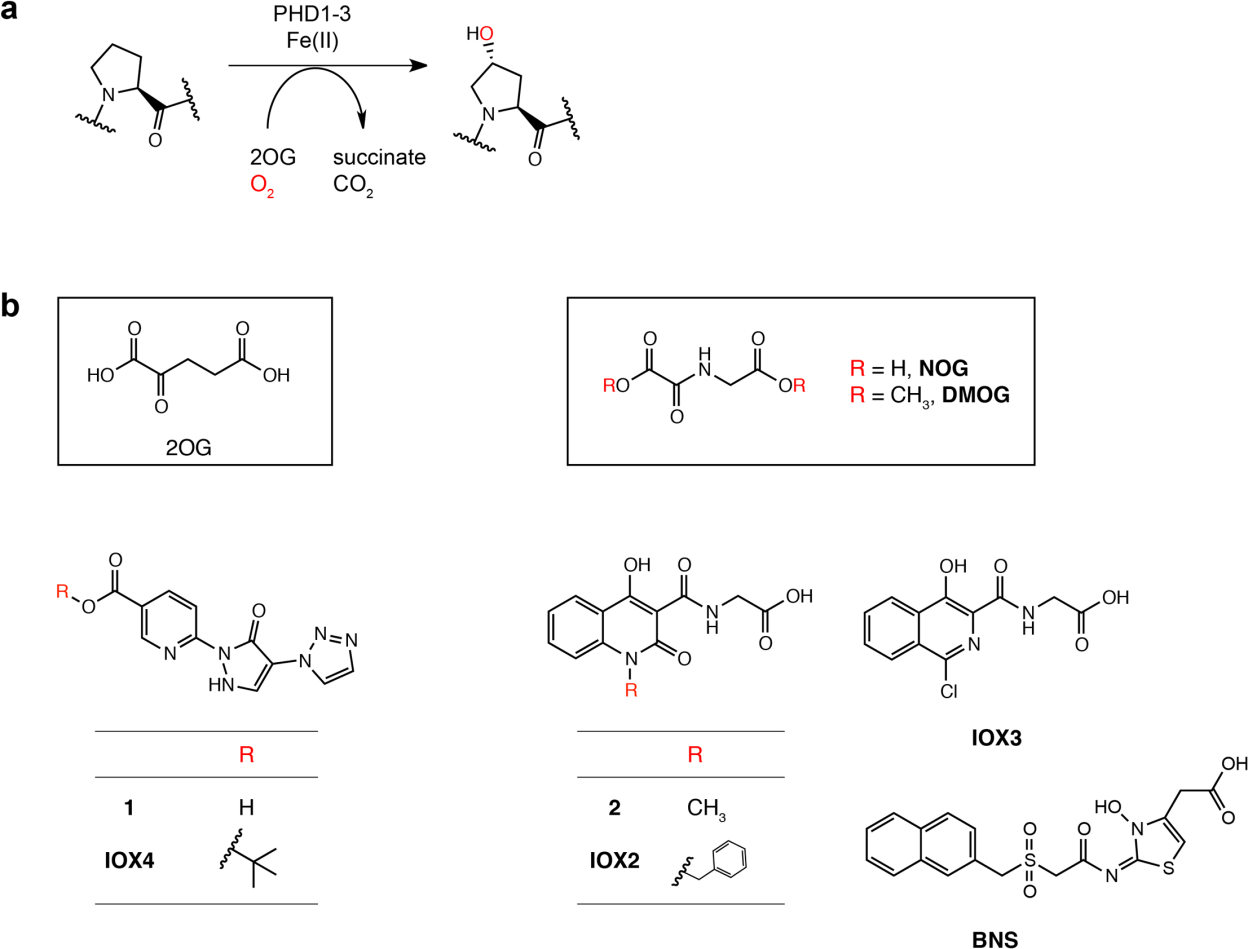
Hydroxylation of HIFα and the chemical structures of IOX4 and other PHD inhibitors used in this study. **(a)** Prolyl-hydroxylation (as catalyzed by the PHDs) of HIFα. **(b)** Structures of the dihydropyrazoles (**1** and **IOX4**) in comparison to structures of 2-oxoglutarate (**2OG**), *N*-oxalylglycine (**NOG**) (a catalytically inactive analogue of 2OG), dimethyloxalylglycine (**DMOG**) (a cell-permeable ester derivative of NOG) and **IOX2** [9]. Chemical structures of previously reported PHD inhibitors (compound **2**, bicyclic isoquinolinyl inhibitor **IOX3** and bicyclic naphthalenylsulfone hydroxythiazole **BNS**) used in this study are also shown.

In metazoans, the α/β heterodimeric hypoxia-inducible factor (HIF) complex activates the expression of hundreds of target genes in response to hypoxia, including those involved in cell growth, apoptosis, energy metabolism and angiogenesis [1]. Prolyl hydroxylation of human HIFα in its *C*- and *N*-terminal oxygen dependent degradation domains (CODD and NODD), as catalyzed by three HIF prolyl hydroxylases (PHD1-3, **Fig 1A**), leads to subsequent HIFα polyubiquitination by the von Hippel-Lindau (VHL) protein complex and proteasomal degradation [2,3,4,5,6] HIFα is also regulated via asparaginyl hydroxylation as catalyzed by factor inhibiting HIF (FIH), a modification which blocks the recruitment of the transcriptional co-activators CBP/p300 to HIFα, so causing reduced HIF transcriptional activity [7,8]. The activities of both the PHDs and FIH are suppressed by limiting oxygen, resulting in HIFα stabilization and activation of the HIF system.

Various compounds have been reported as PHD inhibitors in the academic and patent literature [10,11,12]. Most of these compounds likely bind to the active site iron and compete with 2-oxoglutarate (2OG). Many bind in the pocket occupied by the 2OG (CH_2_)_2_COOH side chain, and as a consequence contain a carboxylic acid, which is often undesirable from a pharmacological perspective. We are interested in identifying potent and selective PHD inhibitors that do not contain a carboxylic acid, including ones that may be useful in treatments for ischemic stroke [13,14]. Recently, a set of tricyclic PHD inhibitor with linked pyridine-carboxylate, dihydropyrazole and triazole rings, such as **1** and **IOX4** (**Fig 1B**) has been reported with one compound being in clinical development [15,16]. This series has the potential for more than one mode of iron chelation and 2OG competition; we were particularly interested in the possibility that the triazole (an established carboxylate mimic), rather than the pyridine-carboxylate (an established 2OG mimic) might occupy the 2OG (CH_2_)_2_COOH binding pocket. To test this proposal, we prepared and studied both the acid (**1**) and tertiary butyl ester (**IOX4**) of the ‘tricyclic series’, both reported as PHD inhibitors, but of uncharacterized selectivity and mechanism of action [15,16].

## Materials and Methods

### Organic synthesis procedures

#### Ethyl 2-(1*H*-1,2,3-triazol-1-yl)acetate and Ethyl 2-(2*H*-1,2,3-triazol-2-yl)acetate [15]

To a solution of 1,2,3-triazole (5.42 g, 78.5 mmol) in acetone (80 mL) at room temperature was added ethylbromoacetate (13.07 g, 80 mmol) and Na2CO3 (11.4 g, 108 mmol). The reaction mixture was heated at 35°C for five days, filtered and then concentrated in vacuo. Flash column chromatography using a gradient of ethyl acetate-hexanes (1–10%) as elutant afforded ethyl 2-(2H-1,2,3-triazol-2-yl) acetate (1.66 g, 14%) and ethyl 2-(1H-1,2,3-triazol-1-yl) acetate (3.56 g, 30%). Spectroscopic data are consistent with those reported [15]. Ethyl 2-(1H-1,2,3-triazol-1-yl) acetate, 1H NMR (400 MHz, CDCl3) δ 7.69 (s, 2H), 5.24 (s, 2H), 4.24 (q, J = 7.15 Hz, 2H), 1.28 (t, J = 7.15 Hz, 3H). Ethyl 2-(2H-1,2,3-triazol-2-yl) acetate 1H NMR (400 MHz, CDCl3) δ 7.75 (s, 1H), 7.71 (s, 1H), 5.19 (s, 2H), 4.26 (q, J = 7.15 Hz, 2H), 1.29 (t, J = 7.15 Hz, 3H).

#### Ethyl 3-(dimethylamino)-2-(1H-1,2,3-triazol-1-yl)acrylate and methyl 3-(dimethylamino)-2-(1H-1,2,3-triazol-1-yl)acrylate [16]

A mixture of ethyl 2-(1H-1,2,3-triazol-1-yl) acetate (336 mg, 2.16 mmol) and DMF·dimethylamine (1.021 g, 6.94 mmol) was placed in a sealed tube and heated at 100°C for 24 hours. The reaction mixture was concentrated in vacuo to yield a mixture (1:3, respectively) of the ethyl 3-(dimethylamino)-2-(1H-1,2,3-triazol-1-yl)acrylate and methyl 3-(dimethylamino)-2-(1H-1,2,3-triazol-1-yl)acrylate (210 mg, 55%). The mixture was used in the next step without further purification. Spectroscopic data are consistent with those reported [16]. Ethyl 3-(dimethylamino)-2-(1H-1,2,3-triazol-1-yl)acrylate, 1H NMR (400 MHz, CDCl3) δ 7.73 (s, 1H), 7.61 (s, 1 H), 7.60 (s, 1H), 4.11 (q, J = 7.0, 2H), 3.06 (br s, 3H), 2.23 (br s, 3H), 1.16 (t, J = 7.0). Methyl 3-(dimethylamino)-2-(1H-1,2,3-triazol-1-yl)acrylate, 1H NMR (400 MHz, CDCl3) δ 7.73 (s, 1H), 7.61 (s, 1 H), 7.60 (s, 1H), 3.63 (s, 3H) 3.06 (br s, 3H), 2.23 (br s, 3H).

#### t-Butyl 6-(5-oxo-4-(1H-1,2,3-triazol-1-yl)-2,5-dihydro-1H-pyrazol-1-yl)nicotinate (IOX4) [16]

To a solution of *t-*butyl-6-hydrazinylnicotinate (1.84 g, 8.8 mmol) in ethanol (16 ml) at room temperature was added the mixture of ethyl and methyl 3-(dimethylamino)-2-(1H-1,2,3-triazol-1-yl)acrylate (1.68 g, 8 mmol assumed) and CF_3_CO_2_H (30 μL). The reaction mixture was stirred in a sealed tube at 80°C for 12 h, then allowed to cool to room temperature. 4M HCl in dioxane (300 μL) was added and the resultant mixture was cooled with an ice-water bath to form a pale yellow paste. The precipitate was washed with ethanol (25 mL). Purification with reverse phase flash column chromatography using a gradient of acetonitrile and water as elutant afforded **IOX4** as a pale yellow solid (761 mg, 29%). Melting point: 194°C. ^1^H NMR (400 MHz, DMSO-d_6_) δ 8.93 (s, 1H), 8.53 (s, 1H), 8.45–8.44 (m, 3H), 7.91 (d, *J* = 1.0 Hz, 1H), 1.57 (s, 9H); ^13^C NMR (101 MHz, DMSO-d_6_) δ 163.2, 148.8, 140.3, 133.3, 124.9, 124.4, 111.5, 81.8, 27.8; Rf = 0.45 (DCM: MeOH: NEt_3_(95: 5: 1)); IR: 3133.11(m) (N-H), 1713.96 (s) (C = O), 1673.59 (s) (C = O), 1611.76 (m) (N-H), 1220.37 (s) (C-O, ester); *m/z* (MS, ES^+^) 296.055 (100%, MNa^+^).

#### 6-(5-oxo-4-(1H-1,2,3-triazol-1-yl)-2,5-dihydro-1H-pyrazol-1-yl)pyridine-3-carbocylic acid hydrochloride [16]

To a solution of t-butyl 6-(5-oxo-4-(1H-1,2,3-triazol-1-yl)-2,5-dihydro-1H-pyrazol-1-yl)nicotinate (50 mg, 0.15 mmol) in CH2Cl2 (0.5 mL) was added CF3CO2H (0.5 mL). The reaction mixture was stirred at room temperature for 1 h and concentrated in vacuo. The residue was suspended in aqueous HCl (1M, 2 mL) and lyophilized. Melting point: ≥316.6°C (decomposed). 1H NMR (500 MHz, D2O) δ ppm 8.84 (s, 1H), 8.29 (d, J = 2.21 Hz, 1H), 8.27 (d, J = 2.05 Hz, 1H), 8.10–8.11 (m, 1H), 7.84–7.84 (m, 1H), 7.79–7.80 (m, 1H). 13C NMR (126 MHz, D2O) δ 173.0, 168.3, 156.8, 151.9, 149.2, 139.6, 137.4, 133.4, 129.5, 126.5, 115.4; IR: 3194.40 (C-OH) (m), 1627 (s) (C = O), 1594.03 (m) (N-H), 1411.93 (s) (aromatic); m/z (MS, ES+) 351.11 (100%, MNa+).

### *In vitro* hydroxylation assays

Inhibition assays for PHD2 (AlphaScreen) [9], the KDMs (AlphaScreen) [17], BBOX [18] and FTO [19] were carried out as previously described. *N*-Terminally biotinylated 19-residue human HIF1α CODD peptide (DLDLEMLAPYIPMDDDFQL) used in the PHD2 inhibition assays was from GL Biochem (Shanghai). IC_50_ values were calculated using nonlinear regression with normalized dose-response fit using GraphPad Prism 5.

#### FIH liquid chromatography—mass spectrometry assay

20 ml FIH enzyme (100 nM) in assay buffer (50 mM Tris.Cl pH 7.8, 50 mM NaCl) was pre-incubated for 15 minutes in the presence of compound and the enzyme reaction was initiated by the addition of 20 ml substrate (200 mM L-ascorbic Acid, 20 mM Fe^2+^, 10 mM consensus sequence ankyrin repeat domain peptide [20,21] [and 20 mM 2-oxoglutarate). After 15 minutes, the enzyme reaction was stopped by the addition of 4 ml 10% formic acid and the reaction mixture transferred to a RapidFire RF360 high throughput sampling robot. Samples were aspirated under vacuum onto a C4 Solid Phase Extraction (SPE) cartridge. After an aqueous wash step (0.1% formic acid in water) to remove non-volatile buffer components from the C4 SPE, peptide was eluted in an organic wash step (85% acetonitrile in water, 0.1% formic acid) onto an Agilent 6530 Q-TOF. Ion chromatogram data was extracted for the non-hydroxylated peptide substrate and the hydroxylated peptide product and peak area data for extracted ion chromatograms were integrated using RapidFire Integrator software. Percentage conversion of substrate to product was calculated in Microsoft Excel and IC_50_ values were calculated using Graphpad Prism 5.

### Cell culture and compound treatment

Human cell lines (RCC4, HeLa, Hep3B, MCF-7 and U2OS) were cultured in DMEM (D6546-500ML; Sigma Aldrich) each supplemented with 10% fetal bovine serum (F7524-500ML; Sigma Aldrich), 2 mM L-glutamine (G7513- 100ML; Sigma Aldrich), 50 units/ml of penicillin, and 50 μg/ml of streptomycin (P0781-100ML; Sigma Aldrich). The RCC4 cell line was a gift from C.H.C.M. Buys (University of Groningen, [2]), the HeLa and Hep3B cell lines were from the European Collection of Cell Cultures (ECACC) [2], the MCF-7 cell line was from the American Type Culture Collection (ATCC) and the U2OS cell line was a gift from S.Galey (ICRF Clare Hall Laboratories, United Kingdom). Cells were treated either with DMSO (control) or compounds (dissolved in DMSO) and added directly into the cell culture medium to the desired final concentration for 4–5 h as previously described [9,22].

### Immunoblotting for cell extracts

Cells were extracted using urea/SDS buffer (6.7 M urea, 10 mM Tris-HCl pH 6.8, 10% glycerol and 1% SDS) and processed for immunoblotting as described [22]. The following primary antibodies were used for immunoblotting: mouse monoclonal HIF1α antibody clone 54 (610958, BD Transduction Laboratories, 1:1000), rabbit polyclonal HIF1α hydroxy-Pro402 antibody (07–1585, Millipore, 1:1000), rabbit monoclonal HIF1α hydroxy-Pro564 antibody clone D43B5 (3434S, Cell Signaling, 1:500), mouse monoclonal HIF1α hydroxy-Asn803 antibody (a kind gift from Dr M. K. Lee, Republic of Korea [23], 1:4000), mouse monoclonal PHD2 antibody clone 76a [24] (1:10), mouse monoclonal FIH antibody clone 162c [25] (1:200) and β-actin/HRP (clone AC15, Abcam). HRP-conjugated swine polyclonal anti-rabbit IgG (P0399, Dako), and goat polyclonal anti-mouse IgG (P0447, Dako) were used as secondary antibodies.

### Quantitative HIF1α meso scale discovery immunoassay

Cells were seeded into 96-well cell culture plates (150 μl culture volume) at approximately 60–70% confluency at least 12 h prior to the compound treatment. Half of the culture medium (75 μl/well) were removed and 75 μl/well of medium containing the desired concentrations of compounds were added into cells for 5 h. The cell medium was then removed and the cells were washed once with phosphate buffered saline (PBS), followed by the addition of lysis buffer (20 mM Tris pH 7.4, 450 mM NaCl, 1 mM EDTA, 1 mM EGTA, 0.5% Triton X-100, 1x complete protease inhibitor cocktail and 100 μM DFO). Plates were then left at 4°C with shaking. Cell extracts (25 μl/well) were added into each well of the MSD Bare Standard Bind 96-well Multi Array plates (L15XA, Meso Scale Discovery) which have been pre-coated for 16 h at 4°C with HIF1α mouse monoclonal antibody (NB100-105, Novus Biologicals, 50 ng/well) and pre-blocked for 1 h at room temperature with 5% skimmed milk. Plates were incubated for 1 h with cell extracts at room temperature with shaking, before being washed thrice with 200 μl/well Tris Wash Buffer (50 mM Tris pH 7.6, 0.5% Tween-20 and 150 mM NaCl) and incubated with 25 μl/well of secondary HIF1α rabbit monoclonal antibody clone EP1215Y (ab51608, Abcam, 1:250) for 1 h at room temperature with shaking. Plates were then washed thrice again with Tris Wash Buffer, followed by incubation with 25 μl/well of MSD SULFO-TAG labeled goat anti-rabbit polyclonal antibody (R32AB-1, Meso Scale Discovery, 25 ng/well). Plates were washed thrice with Tris Wash Buffer, added with 150 μl/well 2X Read Buffer (R92TC, Meso Scale Discovery) before being read with an MSD Sector S600 plate reader (Meso Scale Discovery). Signals generated were normalized to “lysis buffer-only” controls.

### Experiments in mice

All animal experiments were performed on male C57BL/6 mice aged 3 months (purchased from Harlan Laboratories, Blackthorn) housed in specific pathogen free conditions with free access to water and standard chow. Mice were injected intraperitonially with the inhibitor (dissolved in Hanks’ buffered saline solution, pH 7.0, 5% DMSO final) or vehicle at the indicated doses for the indicated time as previously described [26]. Tissues from treated mice were harvested and ‘snap frozen’ with liquid nitrogen before being stored at -80°C. For immunoblotting, a portion of the tissues was removed, weighed and homogenised in urea/SDS buffer before being analysed by immunoblotting with HIF1α rabbit polyclonal antibody (10006421, Cayman Chemical, 1:1000) or HIF2α rabbit polyclonal antibody (PM9 [27], 1:1000). For RNA extraction, tissues were ‘snap frozen’ in liquid nitrogen immediately after harvesting, then ground to a fine powder using a cryogenic tissue pulveriser, keeping samples on dry ice at all times and processed for total RNA extraction using the mirVana miRNA isolation kit (AM1560, Life Technologies) according to the manufacturer’s protocol for processing frozen tissues. All animal procedures were compliant with the UK Home Office Animals (Scientific Procedures) Act 1986 and Local Ethical Review Procedures (University of Oxford Medical Sciences Division Ethical Review Committee).

### Real-time quantitative PCR analyses

Genomic DNA were removed from total RNA preparation using the TURBO DNA-free kit (AM1907, Life Technologies) according to manufacturer’s protocol. Total RNA preparations were reverse-transcribed to cDNA using the High Capacity cDNA kit (4374966, Life Technologies) according to manufacturer’s protocol. SYBR Green-based qRT-PCR were then performed on the synthesised cDNA using Fast SYBR Green Master Mix (4385612, Life Technologies) on an Applied Biosystem StepOnePlus thermocycler (Life Technologies). Relative quantifications were calculated using the ∆∆Ct method normalised to β-actin. The following primers were used: Actb_fwd, CAGCCTTCCTTCTTGGGTATGG; Actb_rev, GAGGTCTTTACGGATGTCAACG; Adm_fwd, CACCCTGATGTTATTGGGTTCA; Adm_rev, TTAGCGCCCACTTATTCCACT; Epo_fwd,; Epo_rev,; Il6_fwd, TGCTGGTGACAACCACGGCC; Il6_rev, GTACTCCAGAAGACCAGAGG; Ldh1_fwd, aggctccccagaacaagatt; Ldh1_rev, TCTCGCCCTTGAGTTTGTCT; Vegfa_fwd, CCACGTCAGAGAGCAACATCA; Vegfa_rev, TCATTCTCTCTATGTGCTGGCTTT.

### Crystallography

Crystals of PHD2 (residues 181–426) in complex with Mn(II)/**1** were grown in sitting drops at 293K using vapor diffusion methods at a protein to reservoir ratio of 1:1. PHD2 protein solution contained 32.0 mg/mL protein (in 50 mM Tris∙HCl pH 7.5), 2.8 mM MnCl_2_ and 2.8 mM **1**. The reservoir solution contained 1.6 M sodium citrate/citric acid pH 6.5 (Hampton Research Crystal Screen-II). Mn(II) was used as a replacement for Fe(II) during crystallisation to prevent metal oxidation and consequent formation of heterogenous sample. The crystals were cryoprotected using well solution that was diluted to 30% v/v glycerol and flash frozen in liquid nitrogen. Data were collected from a single crystal at 100K at the Diamond MX beamline I04-1 (0.9200 Å) equipped with a Pilatus 2M detector. The data were indexed, integrated and scaled using MOSFLM and SCALA [28]. The structure was determined by molecular replacement using PHASER [29] and PDB ID: 4BQY [9] as the search model. Iterative rounds of model building (COOT [30]) and refinement (CNS [31] and PHENIX [32]) were performed until the R and R_free_ converged.

### Modelling

A model predicting the binding mode for PHD2 in complex with Mn(II) and **IOX4** was generated using the PHD2.Mn(II).**1** structure as a template (PDB ID: 5A3U). Parameter and topology files for **IOX4** were generated using PRODRG [33]. The model was conjugate energy minimized using CNS (version 1.3) [31] without applying external energy terms.

### NMR experiments

For water relaxation NMR assays [34,35], apo-PHD2 (50 μM) was used. Solutions were buffered using 50 mM Tris-D11 (pH 7.5) in 12.5% H_2_O and 87.5% D_2_O and 125 mM NaCl. The final concentration of Mn(II) was 50 μM (final volume 160 μL). The water relaxation measurements rely on paramagnetic relaxation enhancement. In our studies, paramagnetic Mn(II) was used as a substitute for the Fe(II) in order to enhance the longitudinal relaxation rate of the bulk water and to stop uncoupled turnover. When an inhibitor is bound to PHD2-Mn(II), it displaces bound water molecules away from the paramagnetic Mn(II) at the PHD2 active site. This leads to a net increase in the bulk water relaxation rate [34,35]. Experiments were recorded using a Bruker AVII 500 MHz instrument equipped with a 5 mm inverse TXI probe, and 3 mm MATCH tubes were used throughout. Saturation recovery (90°_x_−G_1_−90°_y_−G_2_−90°x−G_3_−τ−acq) experiments were performed with 1 scan with a relaxation delay of at least 5 times T_1_ between transients. The gradient pulses were achieved using 1 ms Sinebell gradient pulse (G_1_ = 40%; G_2_ = 27.1%; G_3_ = 15%). The receiver gain was set to minimum value (rg = 1) to prevent receiver overload. Typically, 10−16 delay points varied between 100 ms and 60 s were used. T_1_ values were obtained using the *Bruker T1/T2 relaxation* option and peak area was used for curve fitting. The titrant (typically ∼0.2 μL) was added using a 1 μL plunger-in-needle syringe (SGE), and sample mixing was conducted using a 250 μL gas tight syringe (SGE). Binding constants were obtained by nonlinear curve fitting using OriginPro 8.0 (OriginLab) with the equation previously described [36].

For 2OG displacement assays [37], selective ^1^H-^13^C 1D-HSQC experiments were conducted at 700 MHz using a Bruker Avance III spectrometer equipped with an inverse TCI cryoprobe optimized for ^1^H observation. The CLIP-HSQC sequence was used (without ^13^C decoupling). Typical experimental parameters were as follows: acquisition time 0.58 s, relaxation delay 2 s, number of transients 256−1600. The 1JCH was set to 160 Hz. For the selective version of the experiment, a 6.8 ms Q3 180 degree pulse was used, and selective irradiation was applied at the appropriate [^13^C] chemical shift. Three millimeter MATCH tubes with a 160 μL final sample volume were used. Solutions were buffered using Tris-D11 50 mM (pH 7.5) dissolved in 90% H_2_O and 10% D_2_O. Assays were conducted at 298 K in solutions typically containing 50 μM apo-PHD2, 400 μM Zn(II), 50 μM 1,2,3,4-[^13^C]-2OG or [^13^C]-labeled CODD (uniformly [^13^C]-labeled at proline-564) and 400 μM competitor (except unlabeled CODD competitor used at 800 μM). Selective irradiation was applied at 30.5 ppm for [^13^C]-2OG and 24.25 ppm for [^13^C]-labeled CODD. Percentage displacement was calculated according to equation:

$$\left(I\;-\;I_{0}\right)\;/\;\left(I_{blank}\;-\;I_{0}\right)$$

where *I*_0_ is the intensity of the reporter in the presence of protein but without inhibitor, *I* is the intensity of the reporter in the presence of protein and inhibitor, and *I*_*blank*_ is the intensity of the reporter without protein or inhibitor.

## Results

### Validation of IOX4 as a potent and selective inhibitor of PHD2 *in vitro*

Using an antibody-based (AlphaScreen) *in vitro* hydroxylation assay for PHD2 catalysis [9], both **1** and **IOX4** were found to potently inhibit PHD2 with IC_50_ values of 4.8 nM and 1.6 nM, respectively (**Table 1** and **S1 Fig**). In comparison to previously identified PHD inhibitor **IOX2** (**Fig 1B**, **IOX2** IC_50_ = 22 nM) [9], both **1** and **IOX4** are at least 4-fold more potent *in vitro*. Given that the PHDs are part of the human 2OG-dependent oxygenase family which comprises ~60 members, we investigated the selectivity of **1** and **IOX4** by screening against a panel of human 2OG oxygenases, including the human Jumonji C (JmjC)-domain containing histone demethylases (KDMs), γ-butyrobetaine hydroxylase (BBOX) and the fat mass and obesity associated protein (FTO). The KDM assays utilised analogous AlphaScreen methodology as the PHD2 CODD AlphaScreen assay (with enzyme concentrations within the range of 0.2 to 25 nM) [17], the BBOX assay employed a fluoride-release / detection based fluorescence assay [18], the FTO assay is based on liquid chromatography-mass spectrometry (LC-MS) analysis [19], and the FIH assay is a high-throughput mass spectrometry based assay (Agilent RapidFire LC-MS). Using the IC_50_ values determined from each *in vitro* assay as an approximate measure of selectivity, **1** and **IOX4** are at least 875-fold more selective for PHD2 over all other tested enzymes (**Table 1**). In comparison, **IOX2** displays approximately 400-fold selectivity for PHD2 over the same panel. Note that given the similarity of the catalytic domains of PHD1 and PHD3 to that of PHD2, it is likely that **1** and **IOX4** also potently inhibits PHD1 and PHD3 (as supported by cell based work–see below). Although the panel is incomplete, the results suggest that **1** and **IOX4** are highly selective PHD inhibitors, at least over the 2OG-dependent dioxygenases tested.

**Table 1:** Selectivity profiling of the dihydropyrazoles 1 and IOX4 against a panel of human 2OG-dependent dioxygenases.

	IOX2	NOG	1	IOX4
**PHD2**	0.022 μM [9]	11.2 μM [9]	0.0048 μM	0.0016 μM
**JMJD1A (KDM3A)**	>100 μM [38]	0.17 μM [38]	>20 μM	>20 μM
**JMJD2A (KDM4A)**	100 μM [38]	0.2 μM [38]	>20 μM	>20 μM
**JMJD2C (KDM4C)**	100 μM [38]	0.6 μM [38]	>20 μM	>20 μM
**JMJD2E (KDM4E)**	>100 μM [38]	0.3 μM [38]	>20 μM	>20 μM
**JMJD3 (KDM6B)**	>100 μM [38]	0.14 μM [38]	>20 μM	1.4 μM
**FBXL11 (KDM2A)**	52 μM [38]	15.4 μM [38]	20 μM	20 μM
**JARID1C (KDM5C)**	159 μM [38]	25 μM [38]	>100 μM	>100 μM
**BBOX1**	9.5 μM	0.39 μM [18]	>50 μM	73 μM
**FIH**	> 100 μM	2.3 μM	> 100 μM	23.9 μM
**FTO**	11 μM	4.4 μM [19]	37 μM	49 μM

The IC_50_ values obtained reveal the selectivity of dihydropyrazoles **1** and **IOX4** for PHD2 in comparison with **IOX2** and **NOG**. Assays were carried out as previously reported [9,18,19,38].

PHD2: HIF-prolyl hydroxylase-2, JMJD1A (KDM3A): Lysine-specific demethylase 3A, JMJD2A (KDM4A): Lysine-specific demethylase 4A, JMJD2C (KDM4C): Lysine-specific demethylase 4C, JMJD2E (KDM4E): Lysine-specific demethylase 4E, JMJD3 (KDM6B): Lysine-specific demethylase 6B, FBXL11 (KDM2A): Lysine-specific demethylase 2A, JARID1C (KDM5C): Lysine-specific demethylase 5C, BBOX: γ-butyrobetaine hydroxylase, FIH: factor inhibiting HIF, FTO: fat mass and obesity associated protein.

### IOX4 inhibits PHD2 via binding to the 2OG binding site

**Fig 2:**
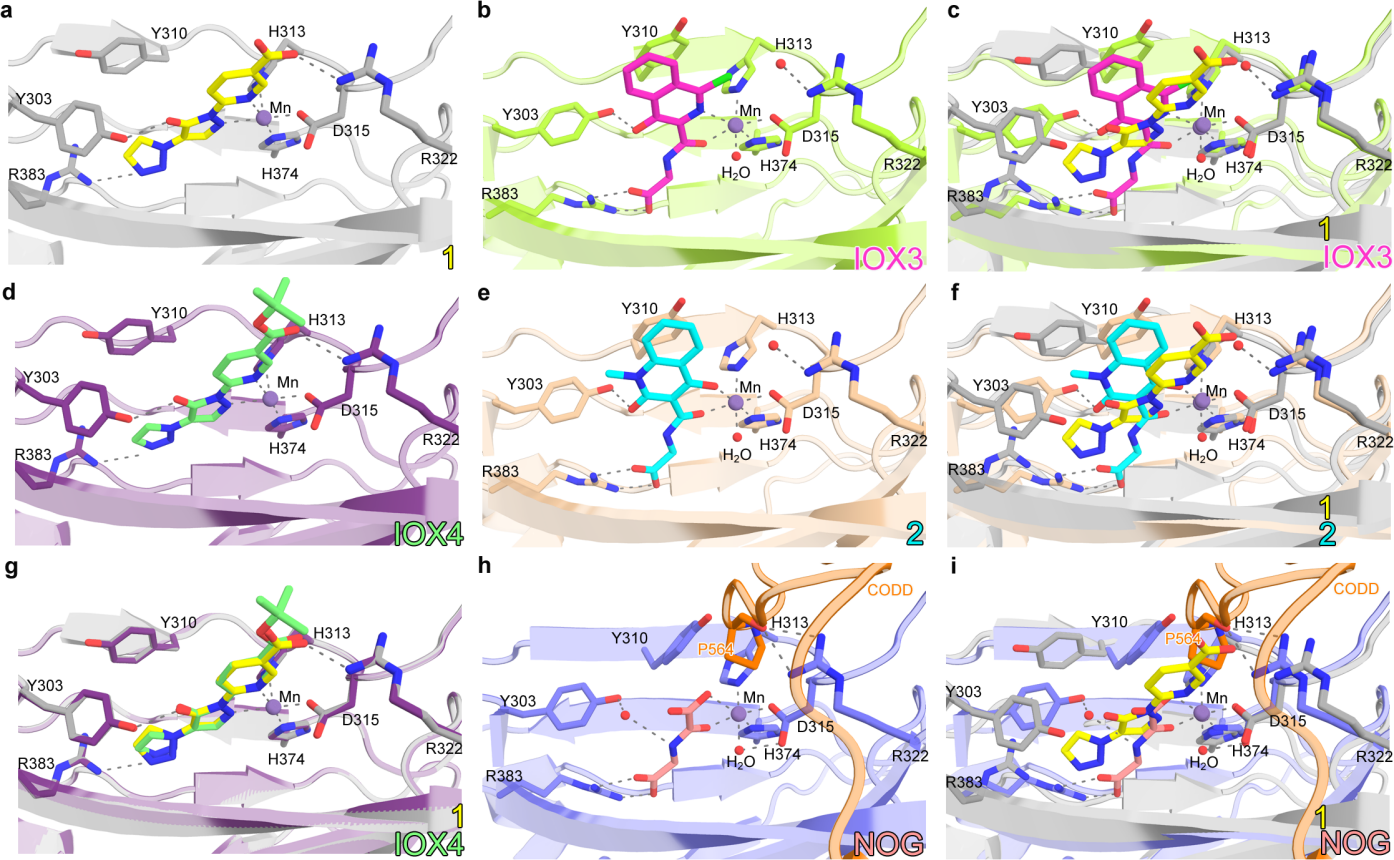
Comparison of the binding modes of PHD inhibitors. Views from crystal structures of PHD2 complexed with **1** (**a**), **IOX3** (**b**), **2** (**e**) and **NOG** (**h**) Compound **1** coordinates the active site metal in a bidentate manner via the nitrogens of its pyridine (*trans* to His374 N*ε*2) and pyrazolone (*trans* to the Asp315 O*δ*1) rings as shown in **a**. A model of **IOX4** binding based on that of **1** (**d**) and the overlay of **a** and **d** (**g**) are shown for comparison. This coordination mode enables **1** to competitively inhibit PHD2 with respect to 2OG (as observed with the other inhibitors described here); the triazole ring of **1** is located in the 2OG C-5 carboxylate binding site whilst the carboxylate side chain of **1** makes electrostatic interaction with another arginine, R322 (1 carboxylate O–NH1 R322, 2.9 Å) that is located at the entrance of the active site; R322 is directly involved in substrate binding (P564/HIF1α CODD O–NH1 R322/PHD2, 2.6 Å; P564/CODD O–NH1 R322/PHD2, 2.8 Å) [39]. Compare **a**, **b** and **c** for differences in binding modes between **1** and **IOX3**; **a**, **e** and **f** for differences between **1** and **2**; **a**, **h** and **i** for differences between **1** and **NOG**. PDB ID: 4BQX (PHD2.IOX3) [9], 4BQW (PHD2.IOX2) [9]; 3HQR (PHD2.NOG.CODD) [39].

To obtain insights into the mode of PHD inhibition by the dihydropyrazoles, we made attempts to crystallize the PHD2 catalytic domain (residues 181–426) in complex with **1** or **IOX4**; a crystal structure of PHD2.Mn(II).**1** was obtained. In the PHD2.Mn(II).**1** complex, the inhibitor coordinates to Mn(II) in a bidentate manner via the nitrogen atoms of its pyridine and pyrazolone rings (**Fig 2A**). In contrast to previously observed binding mode for other PHD inhibitors occupying the 2OG binding site such as **IOX3** (**Fig 2B**) [9], **2** (**Fig 2E**) [9] and **NOG** (**Fig 2H**) [39], the pyridine-based chain of **1** extends towards the entrance of the active site and is positioned to make electrostatic interactions with Arg-322 (which is known to be involved in substrate binding) [39]. The 2OG C-5 carboxylate binding site of PHD2 which is reported to bind a ligand carboxylate in other PHD2.inhibitor complex structures [40,41,42],is occupied by the triazole ring of **1**. Based on modelling studies (**Fig 2D**), **IOX4**, for which we have not obtained a crystal structure, is predicted to bind in an analogous manner to **1**, with its tert-butyl group protruding towards the entrance of the active site.

To further study the mechanism of actions of **1** and **IOX4** in solution, we employed a competition-based nuclear magnetic resonance (NMR) method using labeled 2OG ([^13^C]-2OG) or labeled HIF1α peptide fragment corresponding to the *C*-terminal oxygen-dependent degradation domain ([^13^C]-CODD) as reporter ligands [37]. The results reveal that the previously described inhibitor **IOX2** displaces 2OG from PHD2 (**S3A Fig**), consistent with its predicted binding mode [9]. However, [^13^C]-CODD was not displaced by **IOX2** within limits of detection, suggesting that HIF1α CODD can still bind to PHD2 in the presence of **IOX2**, likely in a non-productive orientation that avoids the steric clash between the **IOX2** benzyl group with the hydroxylated CODD prolyl-residue). Previous crystallographic studies have shown that the HIF substrate can still bind even when the 2OG active site is occupied by some, but not all, inhibitors [39]. Studies with **IOX4** gave similar results to that observed with **IOX2**, with CODD (but not 2OG) remained bound to PHD2.**IOX4** complex, suggesting that **IOX4** competes with and displaces 2OG at the active site of PHD2 (**S3A Fig**). In contrast, a different type of PHD2 inhibitor, i.e. the bicyclic naphthalenylsulfone hydroxythiazole, **BNS** (see **Fig 1B**), was found to displace both 2OG and CODD (**S3A Fig**), consistent with previous observations [35]. Although we did not study NODD inhibition, given that the same active site is responsible for the NODD and CODD hydroxylation [39,40], **1** and **IOX4** are likely to inhibit NODD hydroxylation in a similar manner to that for CODD, as subsequently shown by cellular studies (see below). Together, the crystallographic and NMR-based studies suggest that the dihydropyrazoles **1** and **IOX4** inhibit PHD2 via binding to the 2OG binding site, but do not displace the HIFα substrate of PHD2.

### IOX4 inhibits HIF prolyl hydroxylase activity in cells

**Fig 3:**
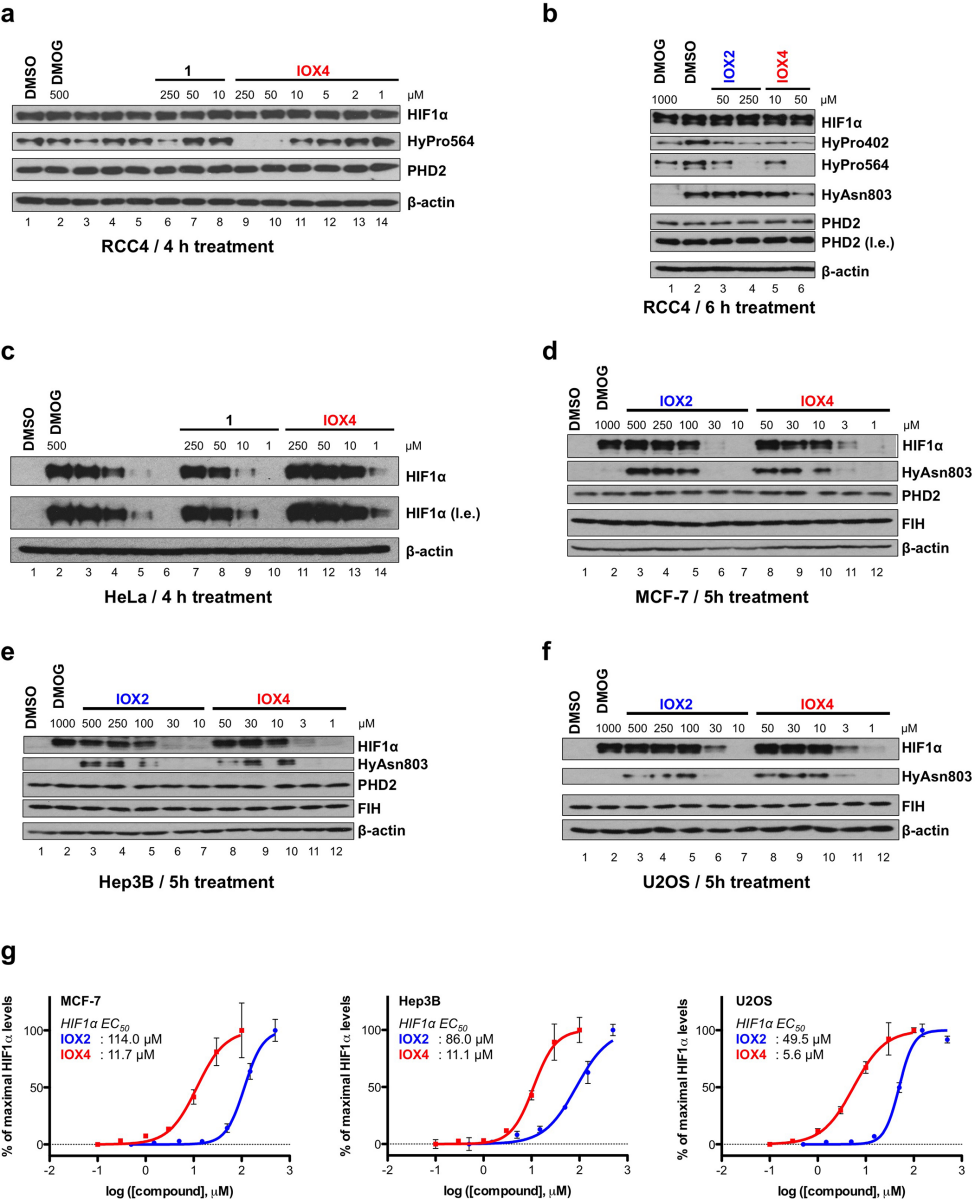
Cellular inhibition of HIF prolyl-hydroxylases by IOX4 leads to HIFα induction. (**a-b**) Immunoblots showing selective inhibition of the HIF1α prolyl-, over asparaginyl-hydroxylation in HIFα-stablized RCC4 cells by **1**, **IOX2** and **IOX4**. (**c**) Immunoblots showing the dose-dependent upregulation of HIF1α in HeLa cells by **1** and **IOX4**. (**d-f**) Immunoblots highlighting the dose-dependent induction of HIF1α in MCF-7 (**d**), Hep3B (**e**) and U2OS (**f**) cells by **IOX2** and **IOX4**. Note the higher potency of **IOX4** compared to **IOX2**, and the lack of inhibition of FIH-catalyzed HIF1α asparaginyl-hydroxylation at concentrations in which maximal HIF1α induction was observed. (**g**) The dose-dependent upregulation of HIF1α in MCF-7, Hep3B and U2OS cells by **IOX2** and **IOX4** as measured using a quantitative HIF1α immunoassay. Each data point represents the average signal ± standard deviation, n = 3. HyPro402: hydroxyproline402; HyPro564: hydroxyproline564; HyAsn803: hydroxyasparagine803; l.e.: long exposure. See **Materials and Methods** for details (including antibodies used).

We then tested the cellular inhibition of **1** and **IOX4** using a von Hippel-Lindau (VHL)-defective human renal cell carcinoma (RCC4) cell line, in which HIFα proteins are constitutively stabilized [22]. In this cell line, the extent of the differential HIF1α prolyl hydroxylation at the *N*-terminal and *C*-terminal oxygen-dependent degradation domains (HyPro402 and HyPro564, respectively), as well as HIF1α asparaginyl hydroxylation at the *C*-terminal transactivation domain (HyAsn803) can be interrogated by immunoblotting using hydroxylation specific antibodies [22,23]. Dimethyloxalylglycine, **DMOG** (a cell-permeable ester of the generic 2OG-dependent dioxygenase inhibitor, **NOG**) was used as a control. The results show that both **1** and **IOX4** block prolyl-hydroxylation of HIF1α at both NODD (HyPro402) and CODD (HyPro564) without markedly affecting the levels of PHD2, consistent with their proposed mode of action via the displacement of 2OG (**Fig 3A and 3B**). **IOX4** was clearly more potent in inhibiting HIF1α prolyl-hydroxylation than **IOX2** (**Fig 3B**); some apparent inhibition of HIF1α asparaginyl-hydroxylation (HyAsn803) was observed at 50 μM of **IOX4**. In VHL-competent HeLa cells, both **1** and **IOX4** induced HIF1α, with **IOX4** being substantially more potent (activity being observed at ≥ 1 μM, **Fig 3C**). Induction of HIF1α levels were observed in MCF-7, Hep3B and U2OS cells treated with **IOX2** and **IOX4**, with **IOX4** apparently being markedly more potent than **IOX2** (**Fig 3D–3F**). These cellular results are consistent with the *in vitro* data indicating that **IOX4** is a substantially more potent PHD inhibitor than **IOX2**.

To quantify the cellular efficiency of PHD inhibition, we developed an electrochemiluminescence-based assay for the quantification of HIF1α levels using the Meso Scale Discovery (MSD, http://www.mesoscale.com) technology (HIF1α MSD assay), which is based on the capture and detection of HIF1α protein using two specific HIF1α antibodies. The technology of this immunoassay is similar to that of the sandwich enzyme-linked immunosorbent assay (ELISA), but utilises electrochemiluminescence-based signal generation and detection [43] (as opposed to the colorimetric-based detection employed in ELISA). The linearity of this assay detection signal was demonstrated using lysates from Hep3B cells treated for 24 h with iron-chelating deferoxamine, DFO to induce HIF1α, with low signal detected in untreated Hep3B cell lysates (**S4 Fig**). Using this assay, we determined the half-maximal effective concentration (EC_50_) for HIF1α induction in three human cell lines (MCF-7, Hep3B and U2OS) after 5 h treatment with **IOX2** or **IOX4**. Consistent with the immunoblotting observations, **IOX4** (HIF1α EC_50_ values = 11.7 μM, 11.1 μM and 5.6 μM in MCF-7, Hep3B and U2OS, respectively) is more potent than **IOX2** (HIF1μ EC_50_ values = 114 μM, 86 μM and 49.5 μM in MCF-7, Hep3B and U2OS, respectively), in all three cell lines at a suitable concentration in cells), with **IOX4** being more potent than **IOX2** in all the tested cell lines.

### HIFα proteins are upregulated in tissues of mice treated with IOX4

**Fig 4:**
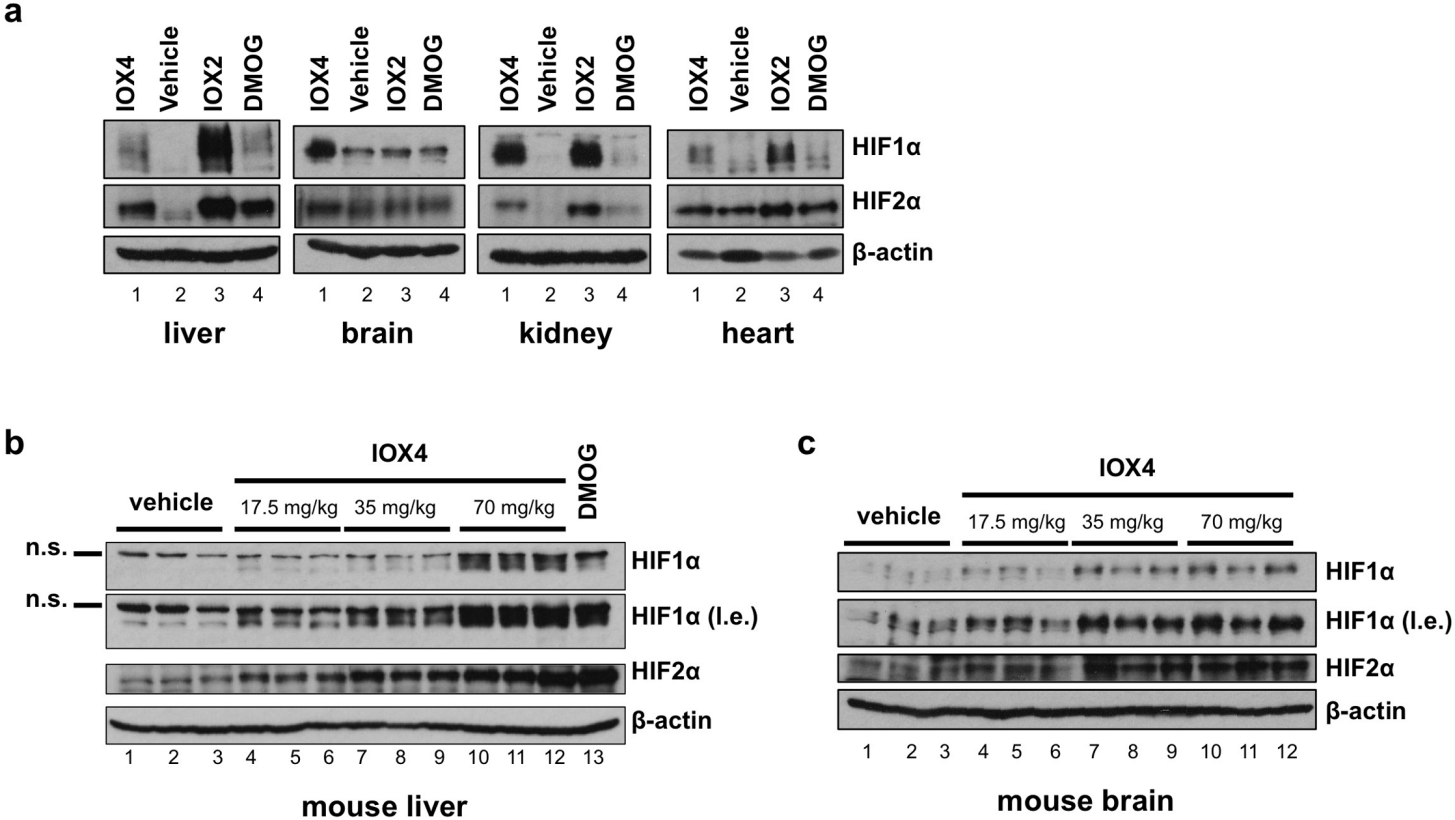
IOX4 induces HIFα in mice. (**a**) Immunoblots showing HIF1α and HIF2α induction in various mouse tissues (liver, brain, kidney, heart) after 1 h treatment at equimolar concentrations of **IOX2** (37.7 mg/kg), **IOX4** (35 mg/kg) or dimethyl *N*-oxalylglycine **DMOG** (75 mg/kg). (**b-c**) Immunoblot showing dose-dependent induction of HIF1α and HIF2α in the mouse liver (**b**) and in the mouse brain (**c**) after 1 h treatment by various doses of **IOX4** (17.5 to 70 mg/kg) in comparison to vehicle control and **DMOG** (160 mg/kg). n.s.: non-specific; l.e.: long exposure.

To explore their utility in a mammalian animal model, **IOX2** and **IOX4** were tested for their ability to induce HIFα in mice. The inhibitors or vehicle controls were injected intraperitonially into wild type C57BL/6 mice and sacrificed after the indicated treatment time before harvesting their tissues to be analysed for HIFα levels by immunoblotting. The results reveal HIF1α is induced in mouse liver after 1 h of treatment of **IOX2**, which persisted even after 2.5 h of treatment albeit at a lower level than the shorter treatment (**S5 Fig**). In contrast, treatment with another PHD inhibitor **FG2216/IOX3** (see **S1B Fig**, previously used in mice studies) [26,44] at an equivalent molar dose to that of **IOX2** led to a lower and shorter induction of HIF1α (**S5 Fig**). To compare the potencies of **IOX2**, **IOX4** and **DMOG**, mice were treated with equimolar concentrations of each inhibitor for 1 h. Immunoblot analyses across multiple tissues reveal induction of HIF1α and HIF2α by all three inhibitors, with **IOX2** displaying the strongest induction followed by **IOX4** and **DMOG** in the liver, kidney and heart (**Fig 4A**). Importantly, both HIF1α and HIF2α were induced in the brain by **IOX4** but not **IOX2** or **DMOG**, suggesting that **IOX4** may better penetrate the blood-brain barrier than the latter two inhibitors. Dose-dependent induction of both HIF1α and HIF2α in the liver and brain was observed after **IOX4** treatment (**Fig 4B and 4C**). These observations reveal both **IOX2** and **IOX4** as active PHD inhibitors in mice; however, induction of HIF1α and HIF2α protein levels in the brain were only observed with **IOX4**.

To investigate whether the induction of HIFα in the mouse brain has an effect on HIF target genes, we performed quantitative real-time PCR (qRT-PCR) analyses on HIF target genes (including those involved in angiogenesis, i.e. *Vegfa*, *Adm* and *Il6*). The analyses reveal that the mRNA levels of some of the HIF target genes tested (*Epo*, *Vegfa* and *Adm*) were induced in a dose-dependent manner after 1 h treatment with **IOX4**, whereas others (*Bnip3*, *Ldha* and *Il6*) remain unaffected (**S6 Fig**). These results indicate that the HIFα proteins induced by **IOX4** are transcriptionally active and could lead to the induction of a subset of HIF target genes.

## Discussion

The combined results reveal **IOX4** as a highly potent and selective inhibitor of human PHD2, As shown by studies with mice, **IOX4** is useful for *in vivo* work; it will then be useful for investigations on the suitability of the PHDs as targets for cerebral diseases such as stroke. The combined kinetic and biophysical analyses reveal that **IOX4** (and **IOX2**) compete with 2OG for binding to PHD2; the triazole rings of the inhibitors bind in the pocket occupied by the CH_2_CH_2_COOH side chain of 2OG. The results thus reveal the potential of non-acid containing PHD inhibitors—an important finding given the potential of HIFα upregulation mediated by PHD inhibition in the treatment of stroke, as acids do not often permeate the blood-brain barrier efficiently.

Notably, the NMR results show that **IOX2** and **IOX4** bind to PHD2 such that they do not, displace the HIF1α substrate from the active site (though they must alter its binding mode / strength of binding). Thus, PHD inhibitors that compete with 2OG may be classified into those that still enable the formation of stable PHD.HIFα complexes (such as *N*-oxalylglycine, **IOX2** and **IOX4**), and those that strongly displace HIFα from the PHDs (such as **BNS** [35]). The physiological consequences of these different modes of inhibition are unclear, but may become apparent with the clinical use of the different categories of PHD inhibitor. **IOX2** and **IOX4** are highly selective PHD inhibitors–this is important given the roles of other 2OG oxygenases; FIH has multiple substrates other than HIFα [45,46,47,48,49,50,51] and the Jumonji-C histone demethylases (KDMs) are generally important in the regulation of gene expression [52].

Studies in human cell lines support the efficacy of **IOX4** as a potent and selective PHD inhibitor, with a maximal level of HIFα induction being observed at low μM concentrations across all the cell lines tested. Although we did not test **IOX4** with PHD1 and PHD3, the potent inhibition of NODD and CODD in cell lines containing all three PHDs [24] implies that **IOX4** also inhibits PHD1 and PHD3, as anticipated given the similarity of the catalytic domain of the three human PHDs [41]. The use of selective antibodies [22] illustrates the selectivity (within limits of detection) of the inhibitors for the PHDs over FIH. It is notable that the concentrations of PHD inhibitors required to maximally induce HIFα vary across the different cell lines. Aside from other considerations (e.g., uptake, export, metabolism), this observation may, in part, reflect the different levels of the PHD isoforms in different cell types [24]. Given that the quantitative control of HIF target genes (e.g., *EPO* and *VEGF*) is of clinical importance in terms of PHD inhibitors use, the effective concentrations and the duration of treatment of any PHD inhibitor are therefore important considerations.

The induction of HIFα in mice treated with **IOX4** reflects the *in vivo* utility of this inhibitor. When compared with the results for **IOX2**, lower HIFα induction by **IOX4** was observed in the liver, heart and kidney; however markedly higher induction by **IOX4** was observed in the brain. As with the variations in the results obtained with different cell lines, the reasons for these organ-specific effects are unclear; in part they likely reflect differences in the transport (uptake/export) and/or metabolism of these inhibitors in the different tissues, but they may also reflect differences in the context dependent regulation of the HIF system. Importantly, **IOX4** is effective at inducing HIFα in the mouse brain–to our knowledge, this is the first selective PHD inhibitor that has been reported to do so in wildtype, uncompromised mice. Although activity in the brain may be an undesirable property of PHD inhibitors aimed at treating anaemia, inhibition of the PHDs is proposed as being protective in ischaemic and hemorrhagic stroke models where it is aimed at inducing blood vessel formation [13,14]. HIF target genes such as *Epo*, *Vegfa* and *Adm* were induced in the mouse brain in a dose-dependent manner after treatment with **IOX4**. However, not all HIF target genes were affected (within limits of detection), as highlighted by the lack of induction of *Bnip3*, *Il6* and *Ldha*. The differing effects in the brain of the PHD inhibitors tested could in part be due to the more hydrophobic nature of **IOX4** compared to **IOX2**, resulting in an improved blood-brain barrier penetration for **IOX4**, though other reasons cannot be ruled out. Note also that there is likely considerable scope for improving the blood brain barrier permeating ability of **IOX4** in terms of lipophilicity and polar surface area [53,54]. The identification of **IOX4** as a selective PHD inhibitor that induces HIFα and its target genes in the brain may thus be a productive step towards validating the PHDs as therapeutic targets for stroke.

## Supporting Information

S1 Table Crystallographic data processing and refinement statistics. (DOCX)

S1 Fig Inhibition of PHD2 activity *in vitro* by IOX4. **(a)** Dose-dependent inhibition of PHD2 activity by the dihydropyrazoles **1** (IC_50_ = 4.8 nM) and **IOX4** (IC_50_ = 1.6 nM) in comparison to **IOX2** (IC_50_ = 22 nM), as determined by the AlphaScreen assay. Each datapoint represents the average ± standard deviation, n≥3.
(TIF)

S2 Fig View of the tPHD2.Mn(II). 1 crystal structure showing the *F*o-*F*c OMIT map (contoured to 3σ) for 1.
(TIF)

S3 Fig NMR studies on the displacement of 2OG and CODD by IOX4. (**a**) Percentage displacement of [^13^C]-2OG and [^13^C]-CODD using an NMR based assay by **IOX2** and **IOX4**. **BNS**, unlabeled CODD and unlabeled NODD were used as controls. Errors shown represent the standard deviation from the means of three separate measurements. (**b**) NMR spectra for the displacement of [^13^C]-2OG and/or [^13^C]-CODD by the PHD inhibitors tested. The positions of the labeled carbon atoms in [^13^C]-2OG are as indicated. [^13^C]-CODD is uniformly labeled at every carbon atom of the highlighted proline residue. (**c**) K_D_ determination for compounds **2** and **IOX2** by a water relaxation-based method. CODD: HIF1α *C*-terminal oxygen-dependent degradation domain, NODD: HIF1α *N*-terminal oxygen-dependent degradation domain. See **Materials and Methods** for assay details.
(TIF)

S4 Fig The detection of HIF1α using the quantitative HIF1α immunoassay based on the Meso Scale Discovery assay technology. Lysates from Hep3B cells ± DFO (24 h treatment) were serially diluted and assayed for the presence of HIF1α using the MSD assay. Each data point represents the average signal ± standard deviation, n = 2.
(TIF)

S5 Fig Induction of HIFα by IOX2 and IOX3 in mice. (**a**) Induction of HIF1α in the mouse liver by **IOX2** (37.7 mg/kg) in comparison to vehicle control, **DMOG** (320 mg/kg) and **IOX3** (30 mg/kg).
(TIF)

S6 Fig Induction of HIFα target genes by IOX4 in mouse brain tissues. Some of the HIF target genes (*Epo*, *Vegfa* and *Adm*) were induced by IOX4 in a dose-dependent manner in the mouse brain tissues after 1 h of treatment. However, other HIF target genes (*Bnip3*, *Ldha* and *Il6)* were not markedly affected.
(TIF)

S7 Fig ^13^C-NMR and ^1^H-NMR for t-Butyl 6-(5-oxo-4-(1H-1,2,3-triazol-1-yl)-2,5-dihydro-1H-pyrazol-1-yl)nicotinate. (TIF)

S8 Fig Synthetic scheme for IOX4. See **Materials and Methods** for details of synthesis.
(TIF)
