# Supplementary material for: Potent and Selective Triazole-Based Inhibitors of the Hypoxia-Inducible Factor Prolyl-Hydroxylases with Activity in the Murine Brain
Source: PLoS One. 2015 Jul 6;10(7):e0132004. doi: 10.1371/journal.pone.0132004 (PMC4492579; doi:10.1371/journal.pone.0132004)
Supplement: S1 Table — (DOCX) [file pone.0132004.s001.docx]

**S1 Table. Crystallographic data processing and refinement statistics**

PDB acquisition code: 5A3U

| **Data Collection** |  |
| --- | --- |
| Space Group | *P*3_2_12 |
| Cell dimensions a,b,c (Å) | 154.94 |
|  | 154.94 |
|  | 85.40 |
| Resolution (Å) | 44.73 – 3.30 (3.48-3.30)* |
| No. of unique reflections | 17598 (2560)* |
| Completeness (%) | 99.2 (99.9)* |
| Redundancy | 6.1 (5.8)* |
| R_sym_** | 0.217 (0.881)* |
| Mean I/σ(I) | 6.7 (1.9)* |
| Wilson *B* value (Å^2^) | 71.6 |
|  |  |
| **Refinement** |  |
| R_factor_^‡^ | 0.202 |
| R_free_ | 0.227 |
| R.m.s. deviation |  |
| Bond length, Å | 0.007 |
| Bond angle, ° | 1.0 |

*Highest resolution shell shown in parenthesis.

**R_sym_ = ∑|*I*-<*I*>|/∑*I*, where *I* is the intensity of an individual measurement and <*I*> is the average intensity from multiple observations.

^‡^R_factor_ = ∑*_hkl_*||*F*_obs_(*hkl*)| − k |*F_c_*_alc_(*hkl*)||/ ∑*_hkl_*|*F*_obs_(*hkl*)| for the working set of reflections; R_free_ is the R_factor_ for ~5% of the reflections excluded from refinement.
