# Supplementary figures and images for: Potent and Selective Triazole-Based Inhibitors of the Hypoxia-Inducible Factor Prolyl-Hydroxylases with Activity in the Murine Brain
Source: PLoS One. 2015 Jul 6;10(7):e0132004. doi: 10.1371/journal.pone.0132004 (PMC4492579; doi:10.1371/journal.pone.0132004)

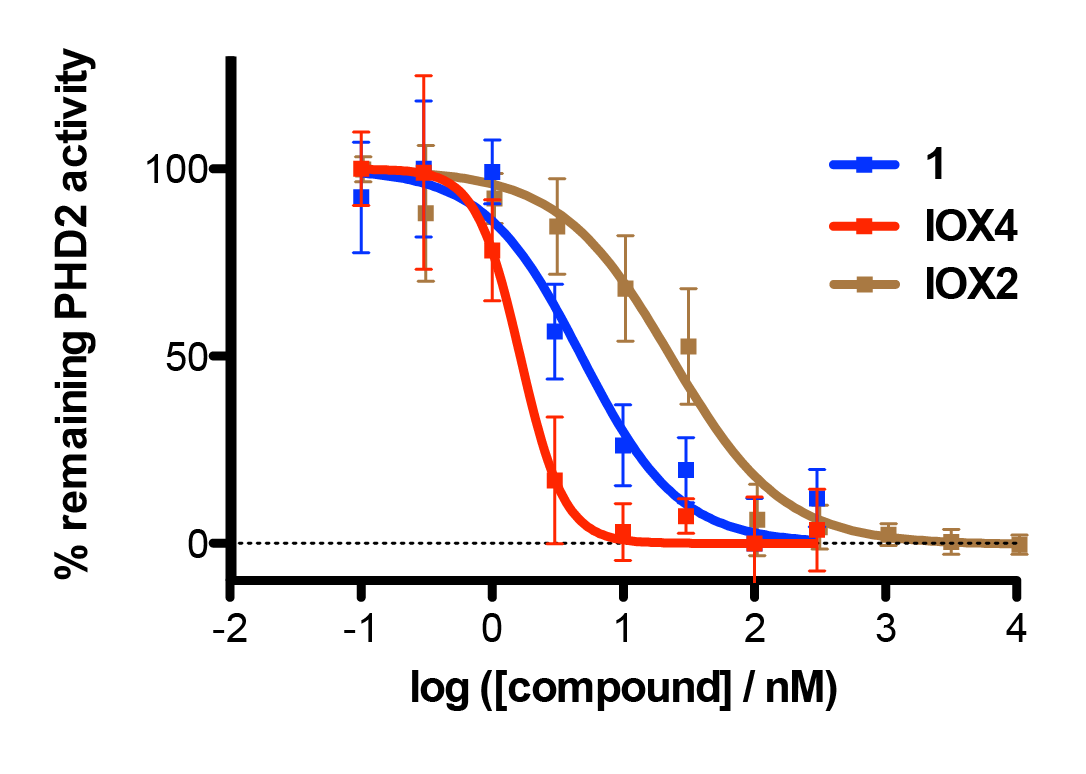

Supplement: S1 Fig — (a) Dose-dependent inhibition of PHD2 activity by the dihydropyrazoles 1 (IC50 = 4.8 nM) and IOX4 (IC50 = 1.6 nM) in comparison to IOX2 (IC50 = 22 nM), as determined by the AlphaScreen assay. Each datapoint represents the average ± standard deviation, n≥3. (TIF) [file pone.0132004.s002.tif]

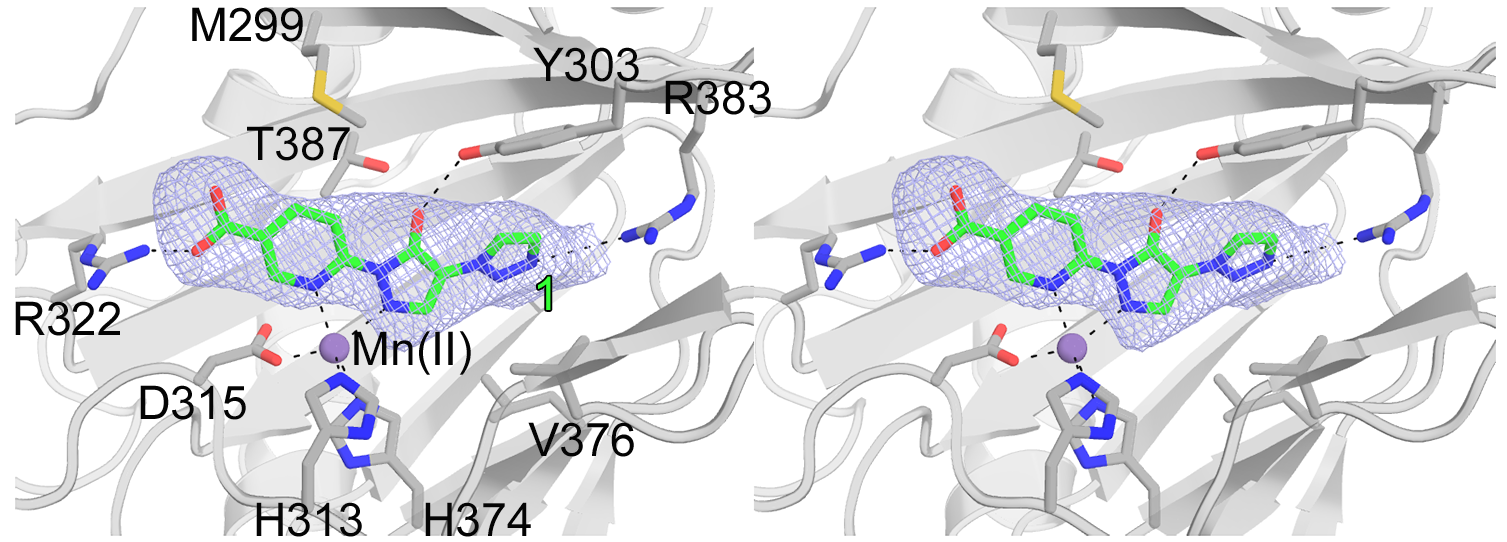

Supplement: S2 Fig — 1 crystal structure showing the Fo-Fc OMIT map (contoured to 3σ) for 1. (TIF) [file pone.0132004.s003.tif]

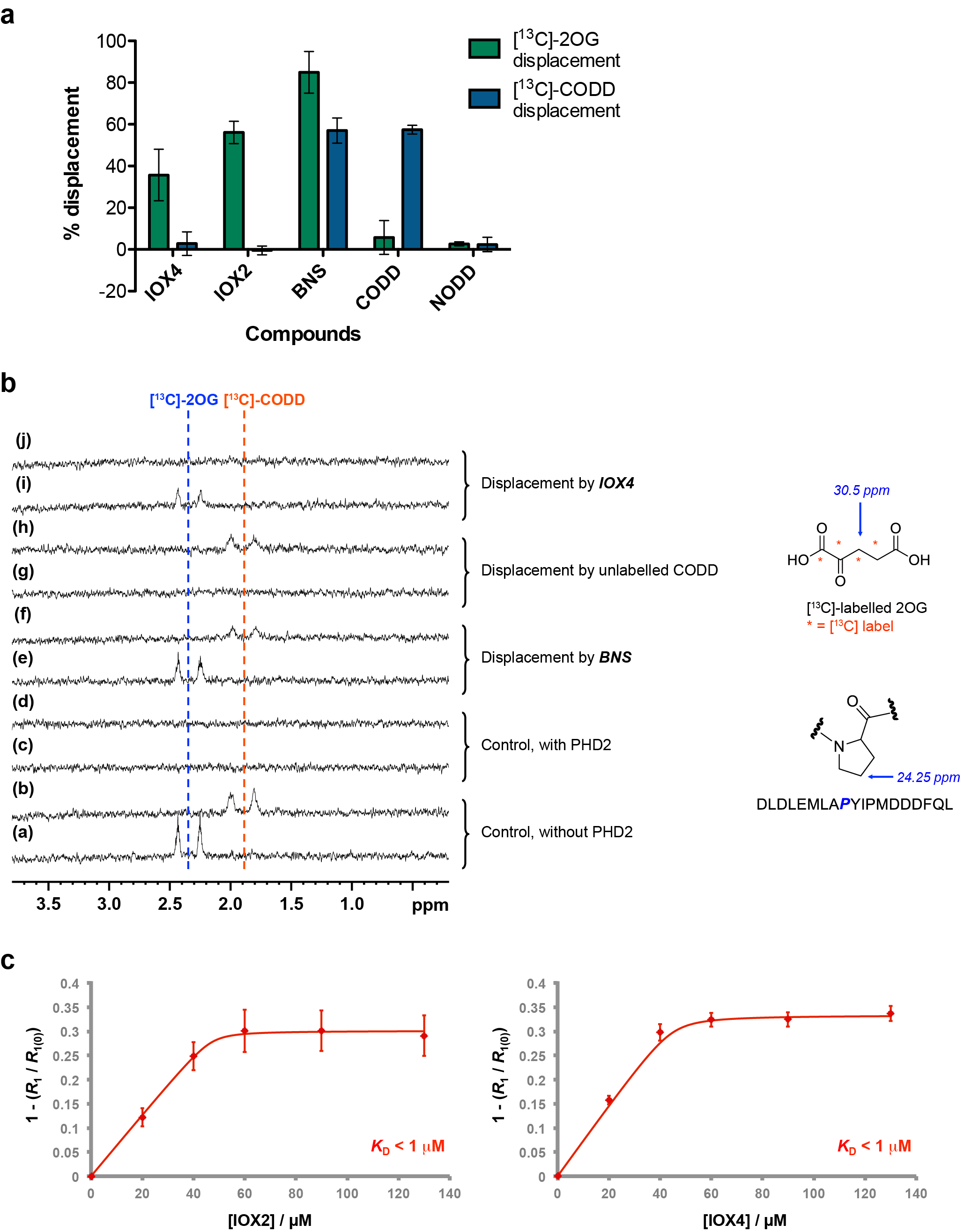

Supplement: S3 Fig — (a) Percentage displacement of [13C]-2OG and [13C]-CODD using an NMR based assay by IOX2 and IOX4. BNS, unlabeled CODD and unlabeled NODD were used as controls. Errors shown represent the standard deviation from the means of three separate measurements. (b) NMR spectra for the displacement of [13C]-2OG and/or [13C]-CODD by the PHD inhibitors tested. The positions of the labeled carbon atoms in [13C]-2OG are as indicated. [13C]-CODD is uniformly labeled at every carbon atom of the highlighted proline residue. (c) KD determination for compounds 2 and IOX2 by a water relaxation-based method. CODD: HIF1α C-terminal oxygen-dependent degradation domain, NODD: HIF1α N-terminal oxygen-dependent degradation domain. See Materials and Methods for assay details. (TIF) [file pone.0132004.s004.tif]

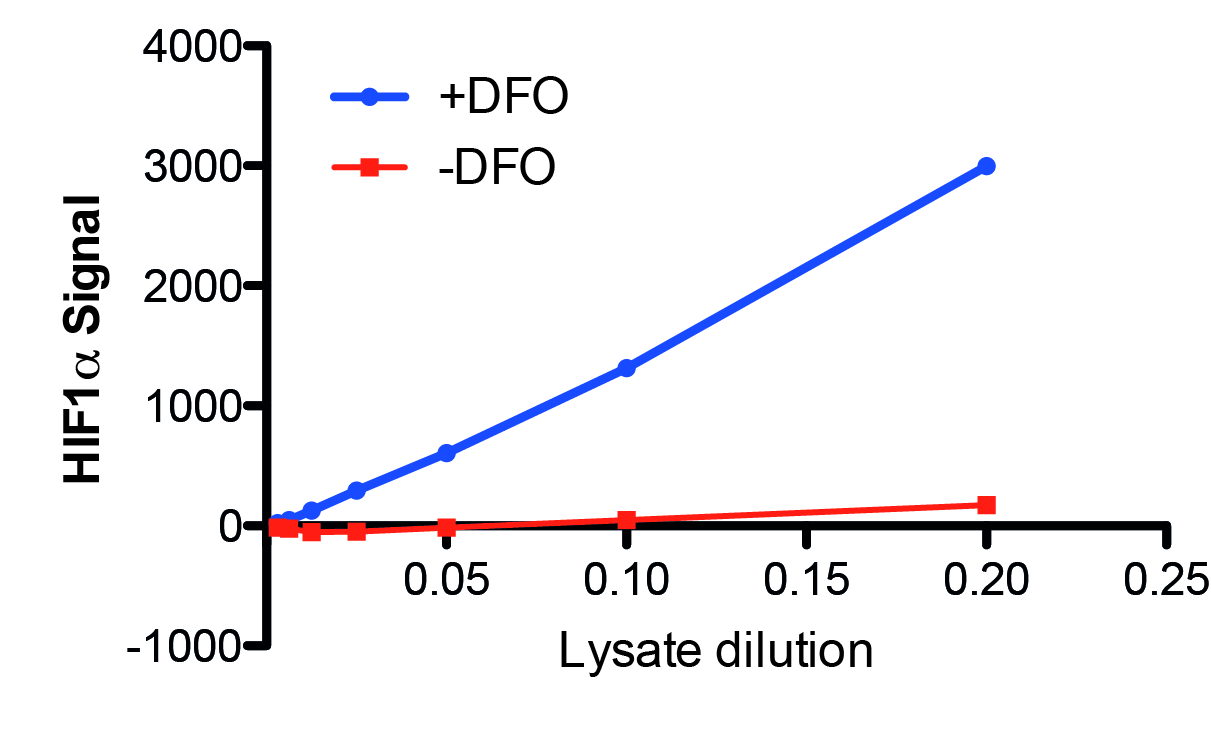

Supplement: S4 Fig — Lysates from Hep3B cells ± DFO (24 h treatment) were serially diluted and assayed for the presence of HIF1α using the MSD assay. Each data point represents the average signal ± standard deviation, n = 2. (TIF) [file pone.0132004.s005.tif]

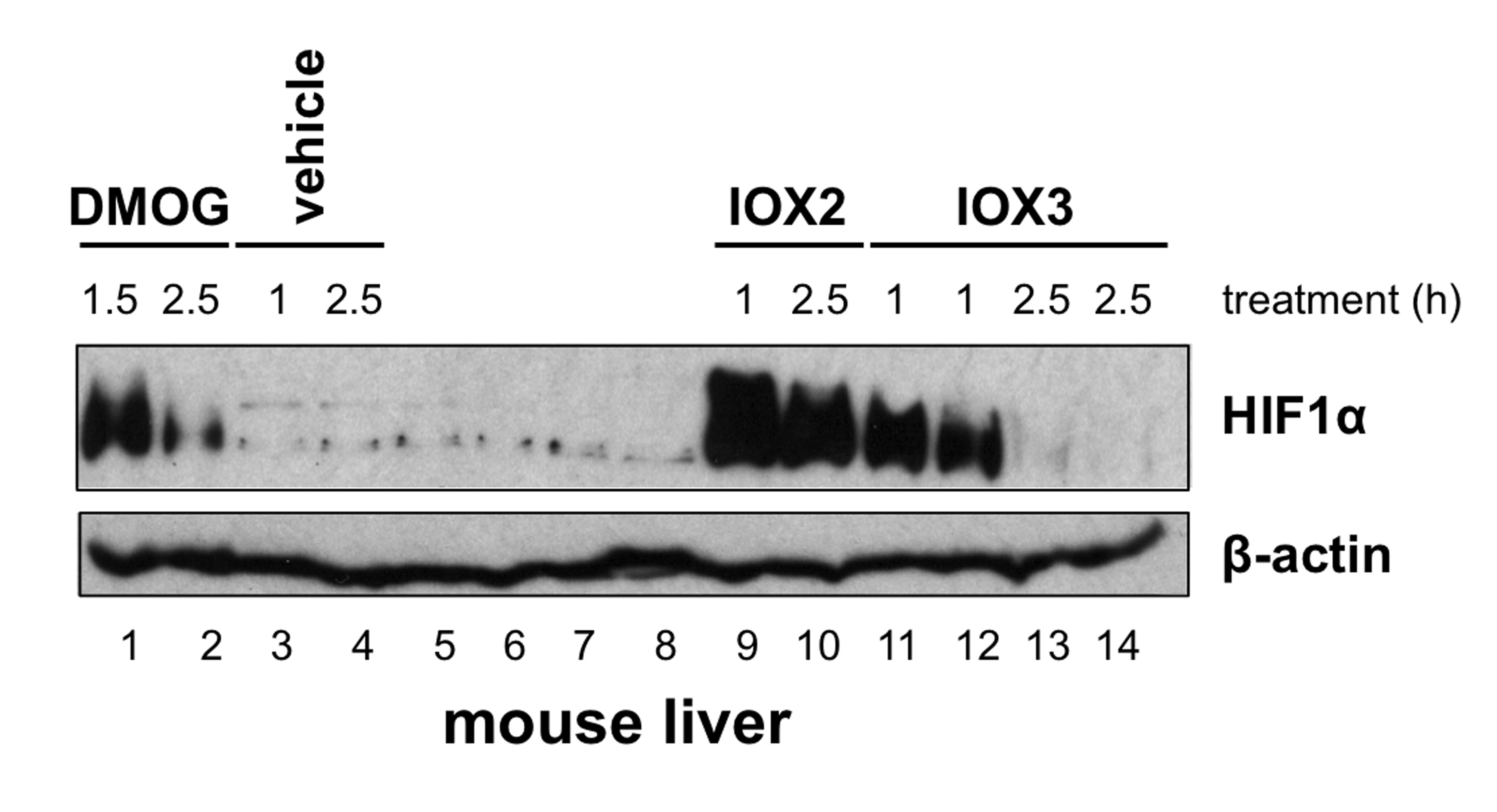

Supplement: S5 Fig — (a) Induction of HIF1α in the mouse liver by IOX2 (37.7 mg/kg) in comparison to vehicle control, DMOG (320 mg/kg) and IOX3 (30 mg/kg). (TIF) [file pone.0132004.s006.tif]

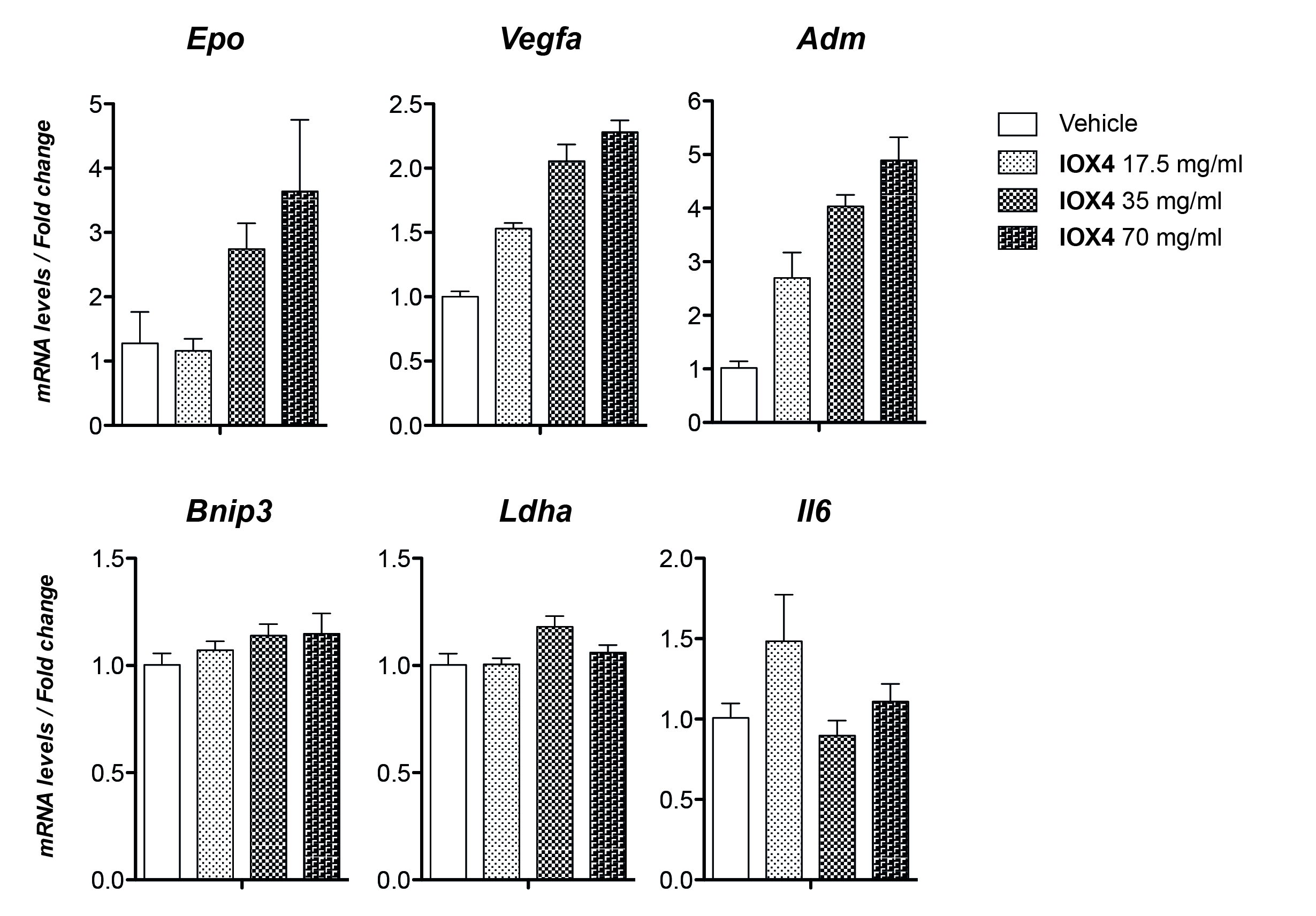

Supplement: S6 Fig — Some of the HIF target genes (Epo, Vegfa and Adm) were induced by IOX4 in a dose-dependent manner in the mouse brain tissues after 1 h of treatment. However, other HIF target genes (Bnip3, Ldha and Il6) were not markedly affected. (TIF) [file pone.0132004.s007.tif]

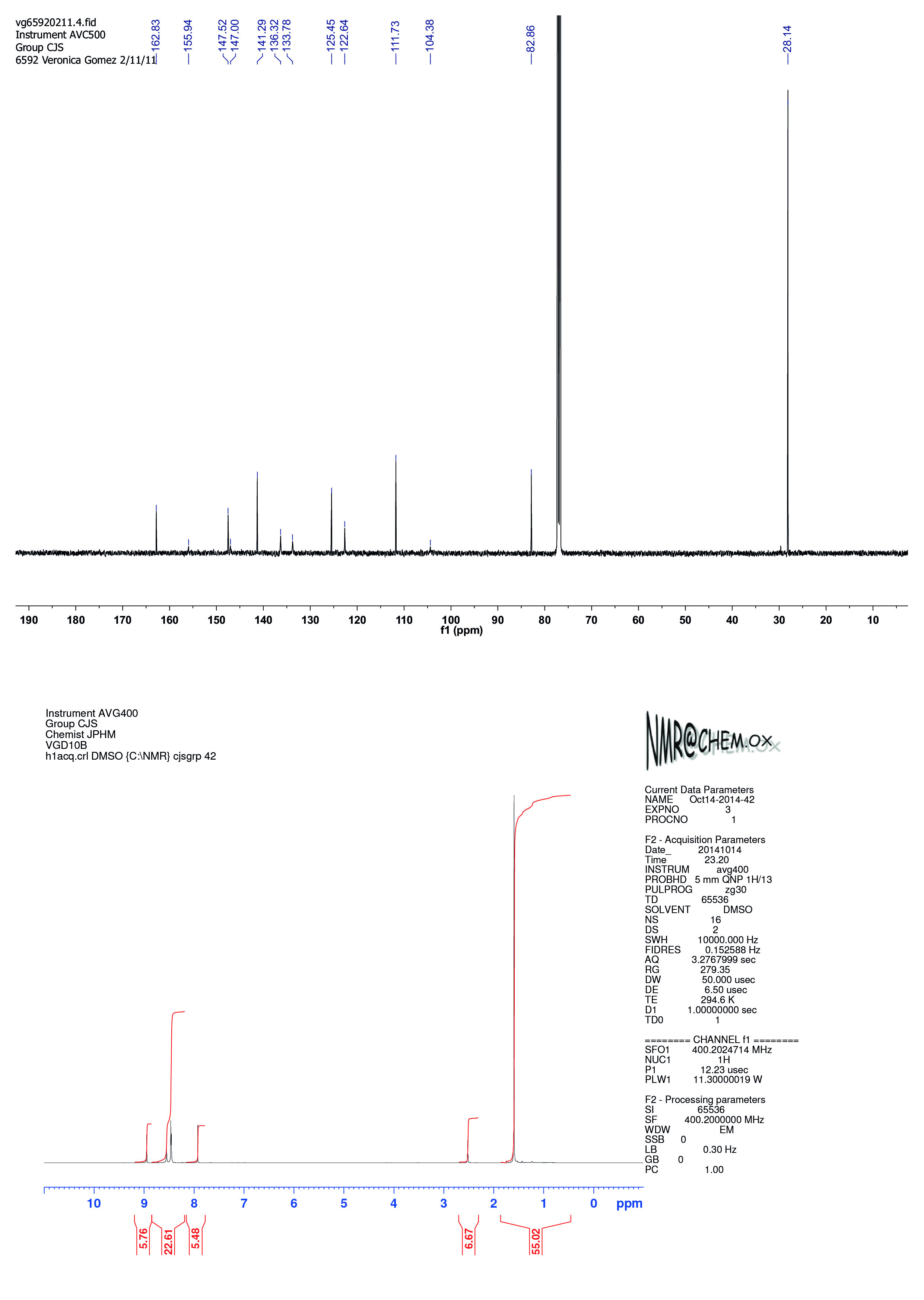

Supplement: S7 Fig — (TIF) [file pone.0132004.s008.tif]

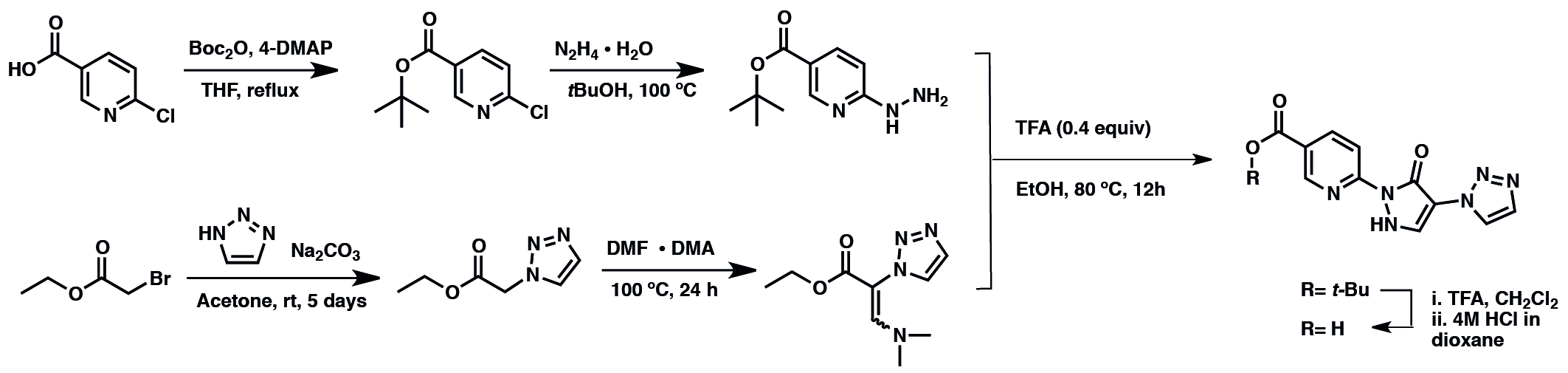

Supplement: S8 Fig — See Materials and Methods for details of synthesis. (TIF) [file pone.0132004.s009.tif]
